# Supplementary material for: Nutritional Status, Dietary Intake, and Adherence to the Mediterranean Diet of Children with Celiac Disease on a Gluten-Free Diet: A Case-Control Prospective Study
Source: Nutrients. 2020 Jan 4;12(1):143. doi: 10.3390/nu12010143 (PMC7019969; doi:10.3390/nu12010143)
Supplement: Supplementary file 1 [file nutrients-12-00143-s001.zip › nutrients-673512-supplementary.docx]

**Supplementary Table S1.** KIDMED – Mediterranean Diet Quality Index in children and adolescents.

|  | **KIDMED test** | **Scoring** |
| --- | --- | --- |
|  | Takes a fruit every day | +1 |
|  | Has a second fruit every day | +1 |
|  | Has fresh or cooked vegetables regularly once a day | +1 |
|  | Has fresh or cooked vegetables more than once a day | +1 |
|  | Consumes fish regularly (at least 2–3 times per week) | +1 |
|  | Goes more than once a week to a fast-food (hamburger) restaurant | -1 |
|  | Likes pulses and eats them more than once a week | +1 |
|  | Consumes pasta or rice almost every day (5 or more times per week) | +1 |
|  | Has cereals or grains (bread, etc.) for breakfast | +1 |
|  | Consumes nuts regularly (at least 2–3 times per week) | +1 |
|  | Uses olive oil at home | +1 |
|  | Skips breakfast | -1 |
|  | Has a dairy product for breakfast (yoghurt, milk, etc.) | +1 |
|  | Has commercially baked goods or pastries for breakfast | -1 |
|  | Takes two yoghurts and/or some cheese (40 g) daily | +1 |
|  | Takes sweets and candy several times every day | -1 |

**Value of the KIDMED index score：**≤ 3: poor adherence to Mediterranean diet**,** 4 to 7: medium adherence to Mediterranean diet**,** ≥ 8: high adherence to Mediterranean diet
